# Mnemonic utilization in stroke education: FAST and BEFAST adoption by certified comprehensive stroke centers

**DOI:** 10.3389/fneur.2024.1359131

**Published:** 2024-03-12

**Authors:** Christopher Hogge, Larry B. Goldstein, Sushanth R. Aroor

**Affiliations:** ^1^Walter Reed National Military Medical Center, Bethesda, MD, United States; ^2^Department of Neurology, College of Medicine, University of Kentucky, Lexington, KY, United States; ^3^Department of Neurology, McGovern Medical School, University of Texas Health Science Center at Houston, Houston, TX, United States

**Keywords:** FAST, BEFAST, stroke, stroke symptoms, public education and awareness

## Abstract

**Introduction:**

Symptom recognition and timely access to treatment are critical components of acute stroke care systems. Two mnemonics widely used in public educational campaigns for recognizing stroke symptoms include FAST (Face-Arm-Speech-Time) and BEFAST (Balance-Eyes-Face-Arm Speech-Time). The FAST mnemonic can miss up to 14% of strokes. BEFAST includes common posterior circulation stroke symptoms and has been implemented by several Comprehensive Stroke Centers (CSCs).

**Methods:**

We sought to analyze the pattern of public educational materials available on the websites of US CSCs. The Joint Commission (JC) quality check website compiles a list containing the names and locations of the country’s 217 JC-certified CSCs, which was downloaded in August, 2022. Each CSC’s website was searched for educational material containing FAST and BEFAST mnemonics for stroke symptom recognition.

**Results:**

The FAST mnemonic was listed by 35% of CSCs, the BEFAST by 58%, with 7% listing no specific mnemonic. The highest portion of CSCs using BEFAST was in western (65%) and southeastern (63%) states. The highest percentage of CSCs with no listed mnemonic were in the northeastern (14%) and southeastern (13%) states.

**Conclusion:**

Consistency is critical in shaping public health education related to stroke symptoms recognition. Our study suggests further effort is needed to unify the public messaging on stroke recognition.

## Introduction

Public education plays an important role in facilitating the prompt recognition of stroke symptoms and ensuring timely access to reperfusion therapy (1). Within educational campaigns, two commonly employed mnemonic aids are FAST (Face-Arm-Speech-Time) and BEFAST (Balance-Eyes-Face-Arm Speech-Time). The FAST mnemonic, although while widely recognized, can still overlook up to 14% of stroke cases, particularly those involving common posterior circulation symptoms (2). While this has led to the adoption of BEFAST mnemonic by many centers, having consistency in public messaging remains paramount. This discrepancy highlights the need to delve into the patterns of mnemonic utilization.

## Methods

The Joint Commission’s quality check website, downloaded in August 2022, contained a list of 217 certified Comprehensive Stroke Centers (CSCs) in the United States, along with their certification status and locations. Each center’s website was then scrutinized for educational material related to stroke symptom identification and the use of either the FAST or BEFAST mnemonic. A Chi-squared test was used to determine statistical significance of the difference between CSCs that used FAST vs. BEFAST, with the null hypothesis being an even distribution of the two mnemonics. *p* < 0.05 was considered significant.

## Results

**Figure 1:**
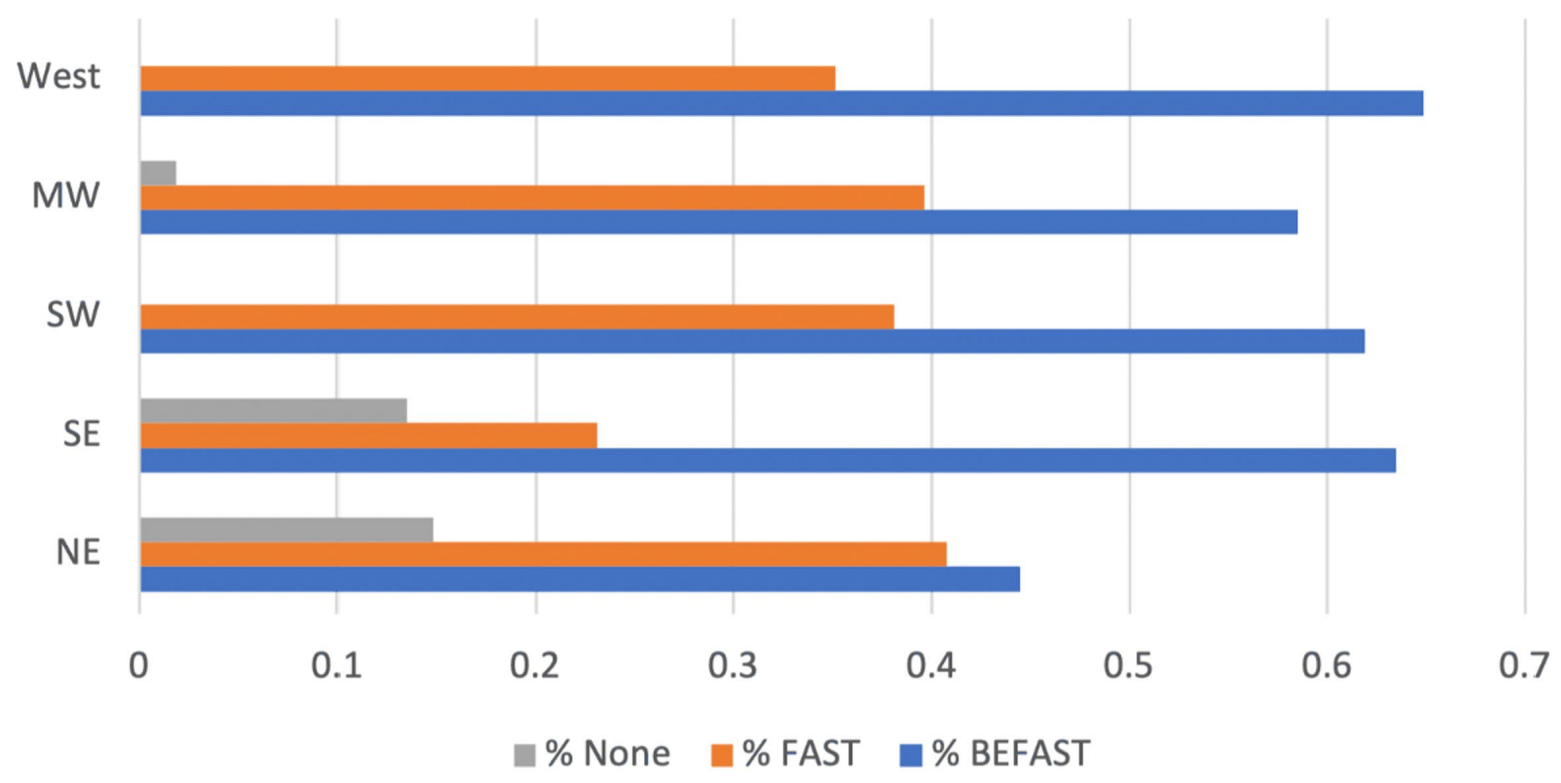
Geographical variation of FAST, BEFAST, or no mnemonic usage by US CSCs in online public educational material.

In total, 93% of Comprehensive Stroke Centers (CSCs) incorporated graphic mnemonics on their websites for public education regarding stroke symptoms. Among these, 35% featured the FAST mnemonic, while 58% opted for the BEFAST mnemonic; notably, 7% listed neither mnemonic (Table 1). The contrast in the proportion of CSCs employing FAST versus BEFAST in their educational material yielded statistical significance (Table 1, *p* = 0.0004). Geographically, the highest adoption rates of BEFAST were observed in the western (65%) and southeastern (63%) states (Figure 1), whereas the northeastern (14%) and southeastern (13%) states had the highest percentage of CSCs without any listed mnemonic (Figure 1).

**Table 1:** Proportion of US CSCs that use FAST, BEFAST, or no mnemonic in online public educational materials.

Mnemonic	Number of CSCs	Percent of total
FAST	76	35.0
BEFAST	125	57.6
No mnemonic	16	7.4
Total	217	100.00

## Discussion

Mnemonic aids serve as invaluable tools for effectively conveying essential public health messages to diverse and extensive populations. Our study underscores that among Comprehensive Stroke Centers (CSCs) in the United States, BEFAST is more frequently used. Noteworthy efforts have been dedicated to promoting a Spanish mnemonic, RAPIDO (3), derived from BEFAST, encompassing “rostro caido” (drooping face), “alteración del equilibrio” (imbalance), “pérdida de fuerza en un brazo o pierna” (loss of strength in an arm or leg), “impedimento visual” (visual impairment), and “dificultad para hablar” (difficulty speaking), emphasizing the imperative of swift action: “obtenga ayuda rápido, llame a emergencias!.” While both the FAST and BEFAST mnemonics have demonstrated efficacy, the inclusion of symptoms associated with vertebro-basilar distribution strokes is vital due to the potential for acute treatment. A systematic review notably underscored BEFAST’s heightened diagnostic value among patients with acute stroke (4). However, it is crucial to acknowledge that BEFAST’s greater sensitivity could also lead to increased recognition of specific stroke mimics (5), potentially affecting the overutilization of health resources and associated costs. Furthermore, it is worth mentioning that no studies have examined the ease of remembering BEFAST. Given the substantial investments in public education campaigns (6), the multifaceted implications of mnemonic selection and its far-reaching effects become even more apparent. This highlights the compelling need for further comprehensive, large-scale studies in public stroke education.

## Data availability statement

The original contributions presented in the study are included in the article/supplementary material, further inquiries can be directed to the corresponding author.

## Author contributions

CH: Data curation, Methodology, Writing – original draft, Writing – review & editing. LG: Writing – original draft, Writing – review & editing. SA: Conceptualization, Supervision, Writing – original draft, Writing – review & editing.
